# Supplementary material for: Unraveling the structure and composition of Varadero Reef, an improbable and imperiled coral reef in the Colombian Caribbean
Source: PeerJ. 2017 Dec 14;5:e4119. doi: 10.7717/peerj.4119 (PMC5733367; doi:10.7717/peerj.4119)
Supplement: Table S1 — List of scleractinian and fire coral species in Varadero and Northern Barú Reefs. Data are the frequency of occurrence (average,%) or presence/absence (+∕ −, visual surveys). [file peerj-05-4119-s001.docx]

| **Species/Family** | **Varadero** | **Baru** |
| --- | --- | --- |
| Family Acroporidae |  |  |
| *Acropora palmata* | + | 0.09 |
| *A. cervicornis* | + | 1.26 |
| *A. prolifera* | + | 0.07 |
|  |  |  |
| Family Agariciidae |  |  |
| *Agaricia agaricites* | 15.86 | 11.26 |
| *A. fragilis* | 0.75 | 0.15 |
| *A. humilis* | + | + |
| *A. undata* | + | 0.17 |
| *A. lamarcki* | + | + |
| *A. tenuifolia* | 12.15 | 4.46 |
| *Agaricia* spp. | 28.76 | 16.04 |
| *Helioseris cucullata* | 0.11 | 0.13 |
|  |  |  |
| Family Poritidae |  |  |
| *Porites astreoides* | 2.58 | 4.41 |
| *P. divaricata* | 1.01 | 2.74 |
| *P. furcata* | 0.73 | 0.33 |
| *P. porites* | 0.53 | 0.96 |
| *Porites* spp. | 2.26 | 4.02 |
|  |  |  |
| Family Siderastreidae |  |  |
| *Siderastrea siderea* | 0.64 | 1.78 |
| *S. radians* | 0.23 | 1.04 |
|  |  |  |
| Family Astrocoeniidae |  |  |
| *Stephanocoenia intercepta* | + | + |
|  |  |  |
| Family Meandrinidae |  |  |
| *Dichocoenia stokesi* | 0.09 | + |
| *Eusmilia fastigiata* | + | + |
| *Meandrina meandrites* | 0.62 | 0.2 |
|  |  |  |
| Family Merulinidae |  |  |
| *Orbicella annularis* | 14.42 | 10.39 |
| *O. faveolata* | 38.06 | 25.57 |
| *O. franksi* | 4.2 | 2.02 |
|  |  |  |
| Family Montastraeidae |  |  |
| *Montastraea cavernosa* | 0.91 | 1.76 |
|  |  |  |
| Family Mussidae, subfamily Mussinae |  |  |
| *Isophyllia sinuosa* | + | 0.13 |
| *I. rigida* | + | -- |
| *Mycetophyllia aliciae* | + | + |
| *M. ferox* | + | + |
| *M. lamarckiana* | + | 0.04 |
| *Scolymia cubensis* | + | + |
| *S. lacera* | + | 0.04 |
|  |  |  |
| Family Mussidae, Subfamiliy Faviinae |  |  |
| *Colpophyllia natans* | 1.78 | 3.07 |
| *Diploria labytinthiformis* | + | 0.85 |
| *Favia fragum* | 0.02 | 0.02 |
| *Pseudodiploria clivosa* | 0.73 | 0.17 |
| *P. strigosa* | 1.49 | 2.5 |
| *Manicina areolata* | + | 0.07 |
|  |  |  |
| Family Oculinidae |  |  |
| *Solenastrea bournoni* | 0.02 | -- |
| *S. hyades* | -- | 0.09 |
| *Oculina diffusa* | + | -- |
|  |  |  |
| Family Pocilloporidae |  |  |
| *Madracis auretenra* | 1.39 | 1.98 |
| *M. decactis* | + | + |
| *M. formosa* | + | + |
|  |  |  |
| Family Milleporidae |  |  |
| *Millepora alcicornis* | 0.85 | 0.28 |
| *M. complanata* | 0.82 | 1.89 |
| *M. striata* | + | -- |
|  |  |  |
| Family Stylasteridae |  |  |
| *Stylaster roseus* | + | + |
