# Supplementary material for: Unraveling the structure and composition of Varadero Reef, an improbable and imperiled coral reef in the Colombian Caribbean
Source: PeerJ. 2017 Dec 14;5:e4119. doi: 10.7717/peerj.4119 (PMC5733367; doi:10.7717/peerj.4119)
Supplement: Table S2 — List of sponge species comparing two reef zones in Varadero and Northern Barú Reefs. Data are frequency of occurrence (%, 30 ×2-m2 transects, n = 7 and 4 at Varadero and Barú, respectively), or presence (+, visual surveys). [file peerj-05-4119-s002.docx]

| Family/Species | Varadero | | | Barú | | | | | |
| --- | --- | --- | --- | --- | --- | --- | --- | --- | --- |
|  | Terrace | Slope | | Terrace | | | Slope | | |
|  | (3-10 m) | (10-24 m) | | (4-13 m) | | | (13-21 m) | | |
| Family Acarnidae |  |  | |  | | |  | | |
| *Acarnus* sp. | + |  | |  | | |  | | |
|  |  |  | |  | | |  | | |
| Family Agelasidae |  |  | |  | | |  | | |
| *Agelas dispar* |  | | + | | |  | | |  |
| *A. sceptrum* | + | + | | |  | | |  | |
| *A. sventres* | 14 |  | | + | | |  | | |
| *A. wiedenmayeri* | 14 | + | |  | | |  | | |
|  |  |  | |  | | |  | | |
| Family Aplysinidae |  |  | |  | | |  | | |
| *Aplysina cauliformis* | 14 | + | |  | | |  | | |
| *A. fulva* | 29 |  | | 25 | | |  | | |
| *Aiolochroia crassa* |  |  | | 25 | | |  | | |
|  |  |  | |  | | |  | | |
| Family Callyspongiidae |  |  | |  | | |  | | |
| *Callyspongia vaginalis* | 14 | + | | 25 | | |  | | |
|  |  |  | |  | | |  | | |
| Family Chalinidae |  |  | |  | | |  | | |
| *Haliclona vansoesti* | 14 |  | |  | | |  | | |
| *Haliclona wallentinae* | 71 | + | | 25 | | |  | | |
|  |  |  | |  | | |  | | |
| Family Chondrillidae |  |  | |  | | |  | | |
| *Chondrilla caribensis* fo. *caribensis* |  |  | | 25 | | |  | | |
|  |  |  | |  | | |  | | |
| Family Clionaidae |  |  | |  | | |  | | |
| *Cliona aprica* |  |  | | 25 | | |  | | |
| *C. laticavicola* | 57 |  | | 75 | | |  | | |
| *C. tenuis* | 29 |  | | 50 | | |  | | |
| *Pione* sp. | 14 |  | | 25 | | |  | | |
|  |  |  | |  | | |  | | |
| Family Coelosphaeridae |  |  | |  | | |  | | |
| *Lissodendoryx colombiensis* | 71 | + | | 75 | | | + | | |
|  |  |  | |  | | |  | | |
| Family Crambeidae |  |  | |  | | |  | | |
| *Monanchora arbuscula* | 71 |  | | 100 | | | + | | |
|  |  |  | |  | | |  | | |
| Family Desmacellidae |  |  | |  | | |  | | |
| *Neofibularia nolitangere* |  |  | | 25 | | |  | | |
|  |  |  | |  | | |  | | |
| Family Dictyonellidae |  |  | |  | | |  | | |
| *Scopalina ruetzleri* | 57 |  | | 75 | | |  | | |
| *Svenzea cristinae* | 29 | + | |  | | |  | | |
| *S. tubulosa* | 14 |  | |  | | |  | | |
|  |  |  | |  | | |  | | |
| Family Dysideidae |  |  | |  | | |  | | |
| *Dysidea etheria* | 14 |  | |  | | |  | | |
|  |  |  | |  | | |  | | |
| Family Halichondriidae |  |  | |  | | |  | | |
| *Halichondriidae* sp. 3 |  |  | | 25 | | |  | | |
| *Hymeniacidon caerulea* | + |  | |  | | |  | | |
| *Topsentia ophiraphidites* | + |  | |  | | |  | | |
|  |  |  | |  | | |  | | |
| Family Halisarcidae |  |  | |  | | |  | | |
| *Halisarca caerulea* | 29 |  | |  | | |  | | |
|  |  |  | |  | | |  | | |
| Family Iotrochotidae |  |  | |  | | |  | | |
| *Iotrochota birotulata* | + |  | |  | | |  | | |
|  |  |  | |  | | |  | | |
| Family Irciniidae |  |  | |  | | |  | | |
| *Ircinia campana* | + | + | |  | | | + | | |
| *I. felix* | 71 | + | | 100 | | | + | | |
| *I. strobilina* |  |  | |  | | | + | | |
|  |  |  | |  | | |  | | |
| Family Microcionidae |  |  | |  | | |  | | |
| *Artemisina melana* |  |  | | 25 | | |  | | |
| *Clathria ?calla* | 29 |  | | 75 | | |  | | |
| *C. curacaoensis* | + |  | |  | | |  | | |
| *C. venosa* |  |  | | + | | | + | | |
|  |  |  | |  | | |  | | |
| Family Mycalidae |  |  | |  | | |  | | |
| *Mycale laevis* | 100 | + | | 100 | | | + | | |
|  |  |  | |  | | |  | | |
| Family Niphatidae |  |  | |  | | |  | | |
| *Amphimedon compressa* | 14 |  | | 25 | | |  | | |
| *Amophimedon viridis* | 14 |  | | + | | |  | | |
| *Niphates ?caycedoi* | 14 |  | | 25 | | |  | | |
| *N. digitalis* |  |  | |  | | | + | | |
| *N. erecta* | 100 | + | | 100 | | | + | | |
|  |  |  | |  | | |  | | |
| Family Petrosiidae |  |  | |  | | |  | | |
| *Neopetrosia carbonaria* | 29 |  | |  | | |  | | |
| *N. rosariensis* |  |  | |  | | | + | | |
| *Petrosia davilai* | + |  | |  | | |  | | |
| *P. pellasarca* |  | + | |  | | |  | | |
| *Xestospongia muta* | + | + | |  | | | + | | |
|  |  |  | |  | | |  | | |
| Family Placospongia |  |  | |  | | |  | | |
| *Placospongia* sp. | 14 |  | |  | | |  | | |
|  |  |  | |  | | |  | | |
| Family Raspailiidae |  |  | |  | | |  | | |
| *Ectyoplasia ferox* |  |  | |  | | | + | | |
|  |  |  | |  | | |  | | |
| Family Suberitidae |  |  | |  | | |  | | |
| *Terpios* sp. | 14 |  | |  | | |  | | |
|  |  |  | |  | | |  | | |
| Family Tedaniidae |  |  | |  | | |  | | |
| *Tedania ignis* |  |  | | + | | |  | | |
| **Total number of species/%^1^** | **36/72** | **14/28** | | **25/50** | | | **dic-24** | | |
| **Number of exclusive species/(%)^2^** | **ago-22** | **feb-14** | | **jul-28** | | | **abr-33** | | |
| **Number of species per transect (min.-max.)** | **10.0 (6-15)** |  | | **10.5 (7-17)** | | |  | | |

^1^ Percent of grand total (50 species)

^2^ Percent of total of each site
