# Supplementary material for: Unraveling the structure and composition of Varadero Reef, an improbable and imperiled coral reef in the Colombian Caribbean
Source: PeerJ. 2017 Dec 14;5:e4119. doi: 10.7717/peerj.4119 (PMC5733367; doi:10.7717/peerj.4119)
Supplement: Table S3 — List of fish species observed at Varadero and Barú. Abundance values are mean (±S.D.) number of individuals per species observed in 30 ×2-m2 belt transects (n = 15 and 7 at Varadero and Barú, respectively). Species observed outside transects are indicated by an x. [file peerj-05-4119-s003.docx]

| **Family/Species** | **Varadero** | | **Barú** | |
| --- | --- | --- | --- | --- |
|  | **Mean** | **±S.D.** | **Mean** | **±S.D.** |
| Family Acanthuridae |  |  |  |  |
| *Acanthurus chirurgus* | 0.07 | 0.26 |  |  |
| *A. coeruleus* | 0.07 | 0.26 | 0.29 | 0.49 |
| *A. tractus* | 0.53 | 1.06 | 0.86 | 1.86 |
|  |  |  |  |  |
| Family Aulostomidae |  |  |  |  |
| *Aulostomus maculatus* | x |  | x |  |
|  |  |  |  |  |
| Family Carangidae |  |  |  |  |
| *Carangoides ruber* | 0.33 | 1.29 | 0.14 | 0.38 |
|  |  |  |  |  |
| Family.Chaetodontidae |  |  |  |  |
| *Chaetodon capistratus* | 0.47 | 0.74 | 0.29 | 0.49 |
| *C. ocellatus* | x |  |  |  |
| *C. striatus* | 0.13 | 0.52 |  |  |
|  |  |  |  |  |
| Family Cirrhitidae |  |  |  |  |
| *Amblycirrhitus pinos* |  |  | 0.14 | 0.38 |
|  |  |  |  |  |
| Family Clupeidae |  |  |  |  |
| *Opisthonema oglinum* | x |  |  |  |
|  |  |  |  |  |
| Family Diodontidae |  |  |  |  |
| *Diodon holocanthus* | 0.80 | 2.31 | x |  |
| *D. hystrix* | x |  |  |  |
|  |  |  |  |  |
| Family Gobiidae |  |  |  |  |
| *Coryphopterus personatus* | 3.07 | 6.20 | 2.57 | 5.94 |
| *Coryphopterus sp.* | 0.07 | 0.26 |  |  |
| *Elacatinus cf. illecebrosus* | 0.13 | 0.35 | 0.14 | 0.38 |
|  |  |  |  |  |
| Family Grammatidae |  |  |  |  |
| *Gramma loreto* | x |  | 0.14 | 0.38 |
|  |  |  |  |  |
| Family Haemulidae |  |  |  |  |
| *Anisotremus virginicus* | x |  | x |  |
| *Haemulon carbonarium* | x |  |  |  |
| *H. flavolineatum* | 1.60 | 1.68 | 1.43 | 1.13 |
| *H. macrostomum* | 0.13 | 0.35 | x |  |
|  |  |  |  |  |
| Family Holocentridae |  |  |  |  |
| *Holocentrus adscensionis* | 0.07 | 0.26 |  |  |
| *H. rufus* |  |  | 0.14 | 0.38 |
| *Myripristis jacobus* | x |  | x |  |
|  |  |  |  |  |
| Family Labridae |  |  |  |  |
| *Bodianus rufus* | 0.33 | 0.49 | 0.43 | 0.79 |
| *Clepticus parrae* | 0.87 | 2.36 | X |  |
| *Halichoeres bivittatus* | 0.20 | 0.56 | 0.57 | 0.79 |
| *H. garnoti* | 0.07 | 0.26 | 0.71 | 1.25 |
| *Thalassoma bifasciatum* | 7.73 | 8.34 | 12.43 | 10.34 |
|  |  |  |  |  |
| Family Lutjanidae |  |  |  |  |
| *Lutjanus analis* |  |  | x |  |
| *L. apodus* | x |  |  |  |
| *L. griseus* |  |  | x |  |
| *L. mahogoni* | x |  |  |  |
| *L. synagris* | x |  |  |  |
|  |  |  |  |  |
| Family Monacanthidae |  |  |  |  |
| *Aluterus scriptus* | x |  |  |  |
|  |  |  |  |  |
| Family Mullidae |  |  |  |  |
| *Mulloidichthys martinicus* | x |  | x |  |
| *Pseudupeneus maculatus* | x |  |  |  |
|  |  |  |  |  |
| Family Muraenidae |  |  |  |  |
| *Echidna catenata* | x |  |  |  |
| *Gymnothorax funebris* |  |  | 0.14 | 0.38 |
|  |  |  |  |  |
| Family Ostraciidae |  |  |  |  |
| *Lactophrys triqueter* | x |  | 0.14 | 0.38 |
|  |  |  |  |  |
| F. Pomacanthidae |  |  |  |  |
| *Holacanthus tricolor* | 0.07 | 0.26 |  |  |
| *Pomacanthus paru* | x |  |  |  |
|  |  |  |  |  |
| Family Pomacentridae |  |  |  |  |
| *Abudefduf saxatilis* | 0.20 | 0.56 | 0.43 | 0.53 |
| *Chromis cyanea* | 0.33 | 0.82 | 0.29 | 0.76 |
| *C. multilineata* | 0.13 | 0.35 | 0.43 | 1.13 |
| *Micropasthodon chrysurus* | 0.33 | 0.82 | 0.86 | 0.69 |
| *Stegastes bipartitus* | x |  |  |  |
| *S. diencaeus* | 8.27 | 5.26 | 13.14 | 5.73 |
| *S. partitus* | 4.53 | 7.21 | 6.57 | 5.35 |
| *S. planifrons* | 10.47 | 6.44 | 9.14 | 6.74 |
|  |  |  |  |  |
| Family Scaridae |  |  |  |  |
| *Scarus iseri* | 8.00 | 4.50 | 12.14 | 4.56 |
| *S. taeniopterus* | 0.13 | 0.52 | 2.00 | 1.53 |
| *Sparisoma aurofrenatum* | 2.00 | 1.20 | 1.57 | 1.13 |
| *S. viridae* | 1.93 | 1.91 | 4.00 | 2.71 |
|  |  |  |  |  |
| F. Sciaenidae |  |  |  |  |
| *Equetus punctatus* | 0.20 | 0.56 |  |  |
| *Odontoscion dentex* | 0.40 | 0.63 | 0.14 | 0.38 |
| *Pareques umbrosus* | x |  |  |  |
|  |  |  |  |  |
| Family Scorpaenidae |  |  |  |  |
| *Pterois volitans* | x |  | x |  |
| *Scorpaena plumieri* | x |  |  |  |
|  |  |  |  |  |
| Family Serranidae |  |  |  |  |
| *Cephalopholis cruentata* | 0.53 | 0.64 | 0.29 | 0.49 |
| *Hypoplectrus puella* | 0.27 | 0.46 | x |  |
| *H. unicolor* | x |  | x |  |
| *Mycteroperca bonaci* | x |  |  |  |
| *Serranus tigrinus* | 0.53 | 0.92 | 1.29 | 1.98 |
|  |  |  |  |  |
| Family Synodontidae |  |  |  |  |
| *Synodus intermedius* |  |  | 0.14 | 0.38 |
|  |  |  |  |  |
| Family Tetraodontidae |  |  |  |  |
| *Canthigaster rostrata* | 0.60 | 0.74 | 1.14 | 0.38 |
| *Spheroides spengleri* | x |  |  |  |
|  |  |  |  |  |
| Family Urobatidae |  |  |  |  |
| *Urobatis jamaicensis* | x |  |  |  |
