# Supplementary figures and images for: Unraveling the structure and composition of Varadero Reef, an improbable and imperiled coral reef in the Colombian Caribbean
Source: PeerJ. 2017 Dec 14;5:e4119. doi: 10.7717/peerj.4119 (PMC5733367; doi:10.7717/peerj.4119)

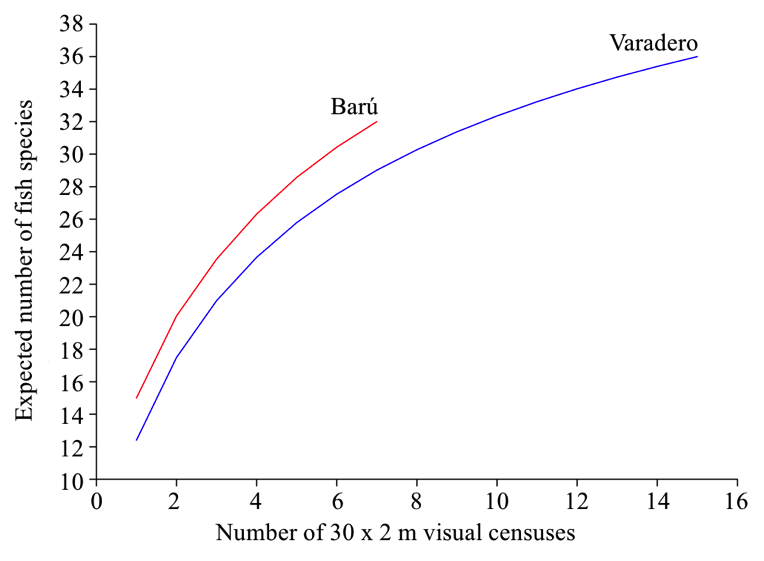

Supplement: Figure S1 — Variation in fish species richness as a function of number of visual censuses (sample-based rarefaction curves) for the fish censuses made at Barú and Varadero Reefs. [file peerj-05-4119-s004.png]
